# Dynamics of soluble vascular endothelial growth factor receptors and their ligands in aqueous humour during ranibizumab for age-related macular degeneration

**DOI:** 10.1186/s12950-018-0203-x

**Published:** 2018-12-04

**Authors:** Ryosuke Motohashi, Hidetaka Noma, Kanako Yasuda, Osamu Kotake, Hiroshi Goto, Masahiko Shimura

**Affiliations:** 1 Department of Ophthalmology, Hachioji Medical Center, Tokyo Medical University, 1163, Tatemachi, Hachioji, Tokyo, 193-0998 Japan; 2 Department of Ophthalmology, Tokyo Medical University, Tokyo, Japan

## Abstract

**Background:**

Intravitreal ranibizumab injection (IRI) is effective for patients with exudative age-related macular degeneration (AMD) and decreases intraocular levels of vascular endothelial growth factor (VEGF), but VEGF receptor intraocular dynamics after IRI are unclear. Therefore, we evaluated changes in the aqueous humor levels of soluble vascular endothelial growth factor receptor (sVEGFR)-1, sVEGFR-2, and their ligands for these receptors (VEGF) patients with AMD receiving IRI.

**Methods:**

The subjects were 24 patients with AMD (24 eyes) who received 3 doses of IRI at monthly intervals. Aqueous humor samples were obtained when each IRI dose was given (visits 0, 1, and 2 at 4-week intervals). Then the suspension array method was employed to measure sVEGFR-1, sVEGFR-2, VEGF, and placental growth factor (PlGF) in aqueous humor samples from the 24 AMD patients and 13 cataract patients (as controls). Best corrected visual acuity (BCVA; logMAR) chart and central macular thickness (CMT; optical coherence tomography) were also assessed over time.

**Results:**

At baseline, the aqueous humor levels of sVEGFR-1, sVEGFR-2, VEGF, and PlGF were significantly higher in the AMD group than in the control group. There was a significant correlation between VEGF and PlGF or between sVEGFR-1 and sVEGFR-2. BCVA and CMT both improved significantly after IRI, and the aqueous humor levels of VEGF, PlGF, and sVEGFR-1 also decreased significantly.

**Conclusions:**

VEGFRs may be involved in the pathogenesis of AMD. IRI improves clinical parameters in AMD patients by suppressing intraocular levels of VEGF, PlGF, and sVEGFR-1.

## Background

Age-related macular degeneration (AMD) is an important cause of visual loss [1]. In neovascular AMD, choroidal neovascularization (CNV) leads to sub-retinal/intra-retinal macular edema, hemorrhage, and fibrosis, resulting in visual impairment. Development of agents targeting vascular endothelial growth factor (VEGF) has advanced considerably since an important role of VEGF in the pathogenesis of CNV was reported [2].

Four anti-VEGF agents are available for AMD, including an aptamer of VEGF (pegaptanib), two anti-VEGF antibodies (bevacizumab and ranibizumab) and a VEGF trapping agent (aflibercept) [3–5]. However, recurrence of CNV after treatment or resistance to these agents is not uncommon; suggesting a role of other factors in CNV along with VEGF. Vitreous fluid levels of soluble VEGF receptor (sVEGFR)-1 and sVEGFR-2 are reported to be elevated in ischemic vitreoretinal diseases [6], and these receptors may have a role in the pathogenesis of macular edema [7]. Interactions between the VEGFRs and their ligands of these receptors (VEGF) may influence progression of AMD. While intravitreal ranibizumab injection (IRI) has been reported to reduce intraocular VEGF [8]. intraocular dynamics of the VEGF receptors after IRI remain unclear.

Accordingly, a prospective study was performed in which the concentrations of VEGFR-1, VEGFR-2, and their ligands (VEGF and placental growth factor (PlGF)) were measured in aqueous humors samples obtained from patients with wet AMD at initiation of IRI, and changes of these factors were evaluated during the first course of IRI treatment.

## Methods

### Subjects

This study was carried out at the Department of Ophthalmology of Tokyo Medical University Hachioji Medical Center (Tokyo, Japan) and was approved by the Ethics Committee of the Center. Study procedures conformed to the Declaration of Helsinki and all patients gave written informed consent to enrollment.

Twenty-four AMD patients (24 eyes) who were scheduled to receive three doses of IRI (Lucentis; 0.5 mg/0.05 ml; Genentech, Inc., South San Francisco, CA) at monthly intervals were studied from July 2013 to March 2015. None of the patients had previously received treatment for AMD. Aqueous humor samples were harvested just before IRI. Control aqueous humor samples were collected during cataract surgery from 13 patients (13 eyes) without other ocular diseases. Best-corrected visual acuity (BCVA) was determined by using a logarithm of the minimum angle of resolution (LogMAR) chart.

Patients with a history of retinal disease other than AMD, glaucoma, uveitis, diabetes, rubeosis iridis, ocular infection, prior laser photocoagulation, or previous intraocular surgery were excluded from the study.

### Fundus findings

In all patients, funduscopy revealed elevated orange-red lesions and/or characteristic polypoidal lesions were found by indocyanine green angiography (IA). Eleven patients had typical wet-type AMD and 13 patients had polypoidal choroidal vasculopathy (PCV) based on the results of fundus examination and IA, but none of them showed retinal angiomatous proliferation (RAP).

Optical coherence tomography (OCT) was performed within 1 week before IRI, employing a spectral-domain apparatus (Spectralis, Heidelberg Engineering, Heidelberg, Germany). The central macular thickness (CMT) was measured with OCT software by two retinal specialists who were blinded to information on the subjects. The severity of macular edema was determined from the CMT.

### Performance of IRI

Each patient received three doses of IRI at 4-week intervals. Just before each injection, 0.1 mL of aqueous humor was harvested by anterior chamber limbal paracentesis using a 30-gauge needle and an insulin syringe. Then IRI was administered through the pars plana at 3.5 mm from the limbus. Immediately after collection, the aqueous humor samples were transferred to sterile plastic tube and stored at − 80 °C until analysis.

### Measurement of cytokines and growth factors

Samples were analyzed by using a suspension array (xMAP; Luminex Corp. Austin, TX) [9, 10] and capture bead kits (Beadlyte; Upstate Biotechnology, Lake Placid, NY) for sVEGFR-1, sVEGFR-2, VEGF, and PlGF according to the manufacturer’s instructions. Standard curves were generated in duplicate for each cytokine by using the cytokine reference set supplied with the kit. To avoid between-run imprecision, we measured the samples from all patients in a single run. Cytokine levels were within the assay detection ranges (minimum detectable concentration was 1.59 pg/ml for sVEGFR-1, 44.81 pg/ml for sVEGFR-2, 0.64 pg/ml for VEGF, and 0.37 pg/ml for PlGF).

### Statistical analysis

Analyses were performed with SAS System 9.4 software (SAS Institute Inc., Cary, North Carolina, USA). Student’s *t*-test was employed to compare normally distributed unpaired continuous variables between the two groups, while the Mann-Whitney U test was used for other variables with a skewed distribution. Differences between the median aqueous levels were assessed with the Wilcoxon single-rank test. To examine relationships among the variables, Spearman’s rank-order correlation coefficients or Pearson’s correlation coefficients were calculated. Dunnet multiple comparison method was also applied. Two-tailed *p* values of less than 0.05 were considered to indicate statistical significance.

## Results

The AMD group (18 men and 6 women) was 76.0 ± 10.6 years old (mean ± SD) and the control group (7 men and 6 women) was 73.1 ± 5.9 years old. There were no significant differences of age (*p* = 0.369) or gender (*p* = 0.189) between two groups.

At the baseline examination (visit 0: just before the first IRI dose), the mean BCVA was 0.39 ± 0.34 in the AMD group and mean CMT was 401 ± 216 μm.

As shown in Table 1, the aqueous humor levels of VEGF, PlGF, sVEGFR-1, and sVEGFR-2 were significantly higher in the AMD group than in the control group (in descending order).

**Table 1:** Aqueous Humor Factors in the Control and AMD Groups

Parameters	AMD Group (*N* = 24)	Control Group (*N* = 13)	*P* value
VEGF (pg/ml)	24.0 [0.64–45.2]	0.64 [0.64–21.6]	0.023
PlGF (pg/ml)	1.59 [0.91–2.82]	0.37 [0.37–2.42]	0.013
sVEGFR-1 (pg/ml)	466 [204–922]	47.1 [47.1–497]	0.004
sVEGFR-2 (pg/ml)	405 [141–636]	104 [44.8–135]	0.003

*AMD* age-related macular degeneration, *VEGF* vascular endothelial growth factor, *PlGF* plancental growth factor, *sVEGFR* soluble vascular endothelial growth factor receptor

In the AMD group, there was a significant correlation between the levels of sVEGFR-1 and sVEGFR-2, as well as between VEGF and PlGF levels (Table 2), but there were no correlations between sVEGFR-1 and VEGF or PlGF (Table 2) or between sVEGFR-2 and VEGF or PlGF (Table 2).

**Table 2:** Correlation Matrix For Aqueous Humor Factors

Variable	VEGF	PlGF	sVEGFR-1	sVEGFR-2
	*r* *P* value	*r* *P* value	*r* *P* value	*r* *P* value
VEGF		0.86 < 0.001	0.18 0.374	−0.03 0.889
PlGF			0.31 0.149	0.01 0.951
sVEGFR-1				0.57 0.006

*VEGF* vascular endothelial growth factor, *PlGF* plancental growth factor, *sVEGFR* soluble vascular endothelial growth factor receptor, *r* correlation coefficient

**Fig. 1:**
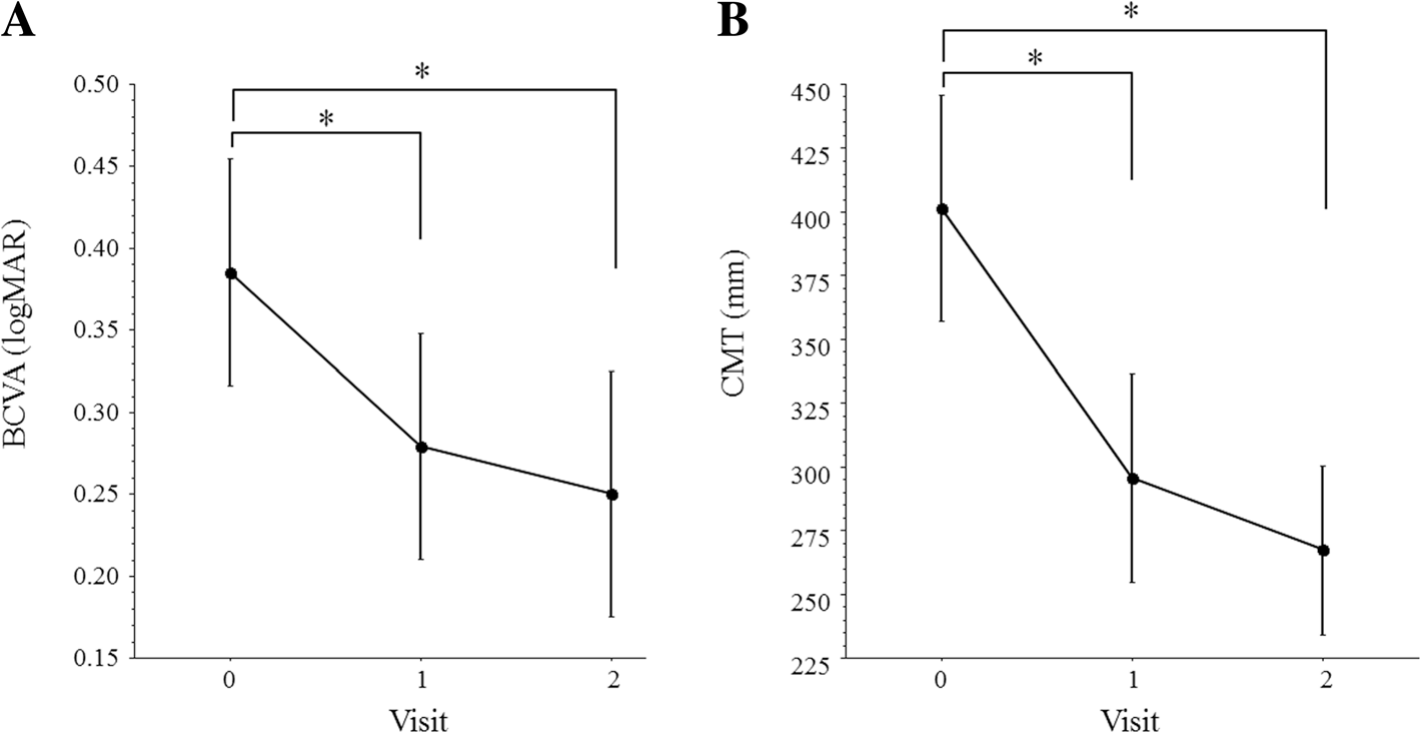
Profile of the mean BCVA and mean CMT after IRI in patients with AMD. **a** Mean BCVA improved significantly from log MAR 0.39 ± 0.34 to log MAR 0.28 ± 0.33 at 1 month (*p* = 0.002) and to log MAR 0.25 ± 0.36 at 2 months (*p* = 0.004). **b** Mean CMT decreased significantly from 401 ± 216 μm to 296 ± 199 μm at 1 month (*p* < 0.001) and to 267 ± 161 μm at 2 months (*P* < 0.001)

In the AMD group, mean BCVA showed significantly imprvement from log MAR 0.39 ± 0.34 before IRI to log MAR 0.28 ± 0.33 after 1 month and log MAR 0.25 ± 0.36 after 2 months (Fig. 1a), while mean CMT decreased significantly from 401 ± 216 μm to 296 ± 199 μm after 1 month and 267 ± 161 μm after 2 months (Fig. 1b).

**Fig. 2:**
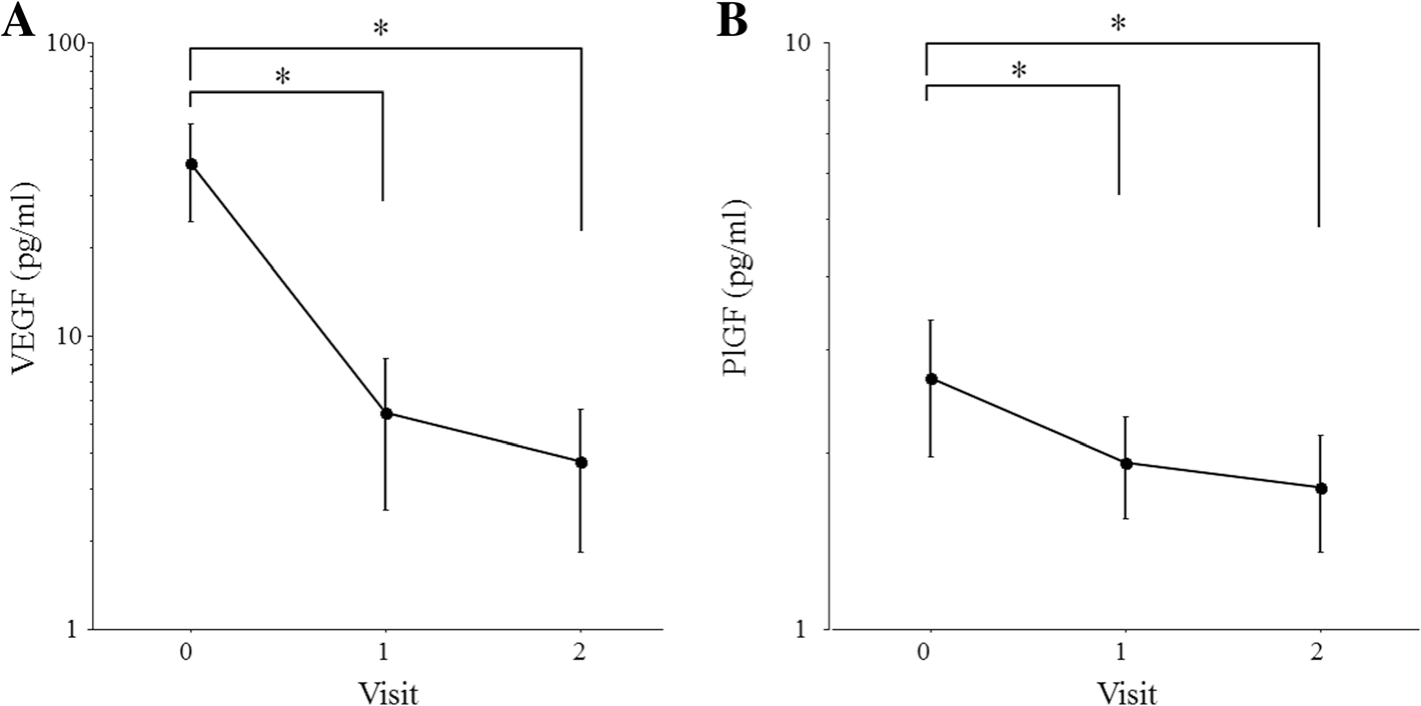
Changes in the aqueous humor level of VEGF or PlGF after IRI in patients with AMD. **a** The aqueous humor level of VEGF showed a significant decrease at 1 month (0.64 pg/ml [0.64–0.64]) and at 2 months (0.64 pg/ml [0.64–0.64]) compared with baseline (24.0 pg/ml [0.64–45.2]) (*P* < 0.001 and *P* < 0.001, respectively). **b** The aqueous humor level of PlGF showed a significant decrease at 1 month (1.27 pg/ml [0.71–2.05]) and at 2 months (1.17 pg/ml [0.48–1.60]) compared with baseline (1.59 pg/ml [0.91–2.82]) (*P* = 0.002 and *P* < 0.001, respectively)

**Fig. 3:**
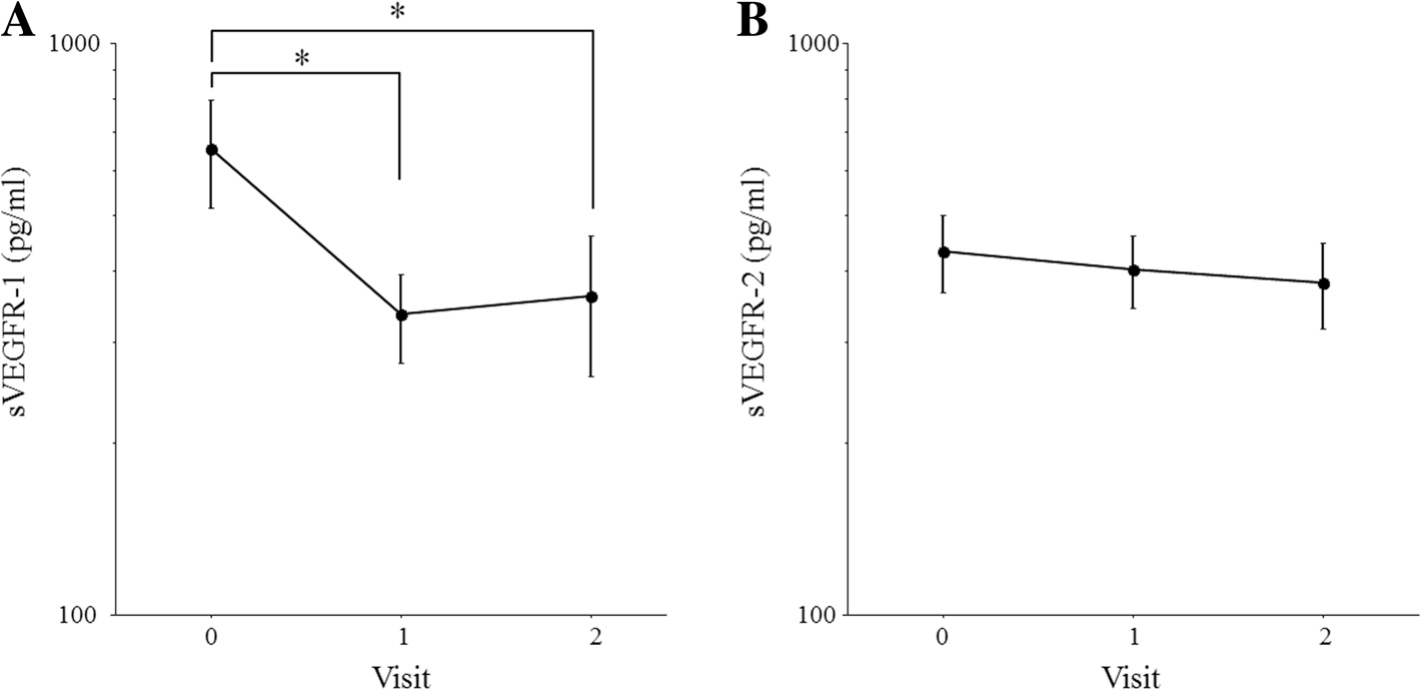
Changes in the aqueous humor level of sVEGFR-1 or sVEGFR-1 after IRI in patients with AMD. **a** Aqueous humor levels of sVEGFR-1 showed a significant decrease at 1 month (268 pg/ml [129–381]) and at 2 months (223 pg/ml [131–326]) compared with baseline (466 pg/ml [204–922]) (*P* = 0.003 and *P* < 0.001, respectively). **b** There was no significant difference in the aqueous humor level of sVEGFR-2 between baseline (405 pg/ml [141–636]) and 1 month (372 pg/ml [144–598]) or 2 months (276 pg/ml [125–643]) (*P* = 0.357 and *P* = 0.303, respectively)

In the AMD group, the aqueous level of VEGF was significantly lower at 1 and at 2 months after starting IRI compared with baseline (Fig. 2a). The aqueous level of PlGF was significantly lower at 1 month and 2 months relative to baseline (Fig. 2b). The aqueous level of sVEGFR-1 was also significantly lower at 1 and at 2 months relative to baseline (Fig. 3a). However, there was no significant change of the aqueous sVEGFR-2 level between baseline and 1 or 2 months (Fig. 3b).

## Discussion

The main new findings were that aqueous levels of both sVEGFR-1 and sVEGFR-2 were significantly higher in the AMD group than the control group, with a significant correlation between sVEGFR-1 and sVEGFR-2 levels, suggesting that expression of these receptors shows concomitant upregulation in AMD. We recently reported [9, 10] that enhanced expression of both transmembrane VEGFR and sVEGFR is involved in regulating angiogenesis and vascular permeability based on the correlation between their expression in human umbilical vein endothelial cells stimulated with phorbol 12-myristate 13-acetate or basic fibroblast growth factor [11]. VEGF signaling may be tightly regulated by VEGFR-1 and VEGFR-2 [12]. VEGF binds to these receptors on vascular endothelial cells, and both receptors have tyrosine kinase activity [13]. VEGFR-1 is also expressed by monocytes/macrophages [14] and promotes VEGF-mediated recruitment of these cells to sites of angiogenesis and inflammation [14, 15]. On the other hand, VEGFR-2 is only expressed by endothelial cells, and VEGF signaling via its membrane-bound isoform influences physiological vascular permeability and angiogenesis [12, 16]. These actions of the VEGF receptors indicate the potential for an important role in the pathogenesis of AMD. Both sVEGFRs were increased in our AMD patients, suggesting that these receptors may be involved in development of CNV, and that the increase of sVEGFRs may be secondary to upregulation of transmembrane VEGFRs.

We also demonstrated that the aqueous humor level of VEGF was significantly suppressed in AMD patients by anti-VEGF therapy, possibly because ranibizumab binds and neutralize intraocular VEGF. These findings are also in agreement with previous reports [8]. IRI also significantly reduced the aqueous humor level of PlGF, which was an unexpected finding VEGF and PlGF exist as dimers in the eye, being either homodimers (VEGF/VEGF and PlGF/PlGF) or heterodimers (VEGF/PlGF) [17, 18]. Therefore, it is possible that the aqueous humor level of PlGF was reduced significantly because ranibizumab bound to and neutralized intraocular VEGF/PlGF heterodimers. Because VEGF acts as a chemoattractant for monocytes after binding to VEGFR-1 and promotes inflammation [19], it is also possible that inflammation was suppressed by ranibizumab binding and neutralizing VEGF. Accordingly, IRI may have reduced inflammation in AMD patients to indirectly down-regulate PlGF expression.

Interestingly, we noted a significant decrease in the aqueous level of sVEGFR-1 after IRI treatment. VEGFR-1 is expressed by monocytes/macrophages [14], and signaling via this receptor promotes VEGF-mediated recruitment of these cells to sites of inflammation [14, 20]. It has been reported that sVEGFR-1 has a pro-inflammatory effect [14, 21], suggesting that elevation of its expression may aggravate inflammation. This idea is supported by the report that sVEGFR-1 expression is correlated with the C-reactive protein level and the leukocyte count [22], which shows a possible link between sVEGFR-1 and inflammation. Chronic inflammation may have a role in the pathogenesis of AMD [23], and VEGF is known to be a chemoattractant for monocytes in primates [24]. Thus, inflammation may be suppressed by ranibizumab binding and neutralizing VEGF and this suppression of inflammation by IRI could be associated with down-regulation of sVEGFR-1 in AMD eyes. On the other hand, there was no significant decrease of sVEGFR-2 expression during IRI, suggesting that its effects are associated with down-regulation of VEGFR-1 rather than VEGFR-2. Because VEGFR-2 signaling mainly influences vascular permeability and angiogenesis [12], the sVEGFR-2 level might not decrease significantly when IRI suppresses inflammation. However, this study had a small sample size and further investigation will be required to clarify the kinetics of sVEGFRs in AMD patients receiving IRI.

According to our findings, improvement of BCVA and CMT occurred during IRI along with reduction of the aqueous humor levels of VEGF, PlGF, and sVEGFR-1. Therefore, IRI may achieve functional and morphological improvement in AMD patients by suppressing intraocular production of VEGF, PlGF, and sVEGFR-1.

## Conclusions

In conclusion, the levels of sVEGFR-1, sVEGFR-2, VEGF, and PlGF in the aqueous humor were significantly higher in patients with AMD than in controls. Aqueous levels of sVEGFR-1 and sVEGFR-2 showed a significant correlation with each other, as did the levels of VEGF and PlGF. IRI significantly improved both BCVA and CMT, while aqueous levels of VEGF, PlGF, and sVEGFR-1 decreased significantly. These findings suggest that VEGFRs may have a role in AMD and that suppression of intraocular VEGF, PlGF, and sVEGFR-1 production by IRI may improve clinical parameters.

## Abbreviations

AMD: Age-related macular degeneration
BCVA: Best-corrected visual acuity
CMT: Central macular thickness
CNV: Choroidal neovascularization
IA: Indocyanine green angiography
IRI: Intravitreal ranibizumab injection
logMAR: Logarithm of minimal angle of resolution
OCT: Optical coherence tomography
PCV: Polypoidal choroidal vasculopathy
PlGF: Placental growth factor
RAP: Retinal angiomatous proliferation
sVEGFR: Soluble VEGF receptor
VEGF: Vascular endothelial growth factor

## References

[1] Jager RD, Mieler WF, Miller JW (2008). Age-related macular degeneration. N Engl J Med.

[2] Rosenfeld PJ, Brown DM, Heier JS, Boyer DS, Kaiser PK, Chung CY, Kim RY (2006). Ranibizumab for neovascular age-related macular degeneration. N Engl J Med.

[3] Carmeliet P, Moons L, Luttun A, Vincenti V, Compernolle V, De Mol M (2001). Synergism between vascular endothelial growth factor and placental growth factor contributes to angiogenesis and plasma extravasation in pathological conditions. Nat Med.

[4] Holash J, Davis S, Papadopoulos N, Croll SD, Ho L, Russell M (2002). VEGF-Trap: a VEGF blocker with potent antitumor effects. Proc Natl Acad Sci U S A.

[5] Heier JS, Antoszyk AN, Pavan PR, Leff SR, Rosenfeld PJ, Ciulla TA (2006). Ranibizumab for treatment of neovascular age-related macular degeneration: a phase I/II multicenter, controlled, multidose study. Ophthalmology.

[6] Matsunaga N, Chikaraishi Y, Izuta H, Ogata N, Shimazawa M, Matsumura M, Hara H (2008). Role of soluble vascular endothelial growth factor receptor-1 in the vitreous in proliferative diabetic retinopathy. Ophthalmology.

[7] Noma H, Mimura T, Eguchi S (2013). Association of inflammatory factors with macular edema in branch retinal vein occlusion. JAMA Ophthalmol.

[8] Wang X, Sawada T, Kakinoki M, Miyake T, Kawamura H, Saishin Y, Liu P, Ohji M (2014). Aqueous vascular endothelial growth factor and ranibizumab concentrations after monthly and bimonthly intravitreal injections of ranibizumab for age-related macular degeneration. Graefes Arch Clin Exp Ophthalmol.

[9] Noma H, Mimura T, Yasuda K, Shimura M (2014). Role of soluble vascular endothelial growth factor receptors-1 and -2, their ligands, and other factors in branch retinal vein occlusion with macular edema. Invest Ophthalmol Vis Sci.

[10] Noma H, Mimura T, Yasuda K, Shimura M (2015). Role of soluble vascular endothelial growth factor receptor signaling and other factors or cytokines in central retinal vein occlusion with macular edema. Invest Ophthalmol Vis Sci.

[11] Hornig C, Barleon B, Ahmad S, Vuorela P, Ahmed A, Weich HA (2000). Release and complex formation of soluble VEGFR-1 from endothelial cells and biological fluids. Lab Investig.

[12] Shibuya M (2006). Differential roles of vascular endothelial growth factor receptor-1 and receptor-2 in angiogenesis. J Biochem Mol Biol.

[13] de Vries C, Escobedo JA, Ueno H, Houck K, Ferrara N, Williams LT (1992). The fms-like tyrosine kinase, a receptor for vascular endothelial growth factor. Science.

[14] Clauss M, Weich H, Breier G, Knies U, Rockl W, Waltenberger J, Risau W (1996). The vascular endothelial growth factor receptor Flt-1 mediates biological activities. Implications for a functional role of placenta growth factor in monocyte activation and chemotaxis. J Biol Chem.

[15] Luttun A, Tjwa M, Moons L, Wu Y, Angelillo-Scherrer A, Liao F (2002). Revascularization of ischemic tissues by PlGF treatment, and inhibition of tumor angiogenesis, arthritis and atherosclerosis by anti-Flt1. Nat Med.

[16] Shalaby F, Rossant J, Yamaguchi TP, Gertsenstein M, Wu XF, Breitman ML, Schuh AC (1995). Failure of blood-island formation and vasculogenesis in Flk-1-deficient mice. Nature.

[17] DiSalvo J, Bayne ML, Conn G, Kwok PW, Trivedi PG, Soderman DD, Palisi TM, Sullivan KA, Thomas KA (1995). Purification and characterization of a naturally occurring vascular endothelial growth factor.Placenta growth factor heterodimer. J Biol Chem.

[18] Yang X, Zhang Y, Yang Y, Lim S, Cao Z, Rak J, Cao Y (2013). Vascular endothelial growth factor- dependent spatiotemporal dual roles of placental growth factor in modulation of angiogenesis and tumor growth. Proc Natl Acad Sci U S A.

[19] Shibuya M (2011). Vascular endothelial growth factor (VEGF) and its receptor (VEGFR) signaling in angiogenesis: a crucial target for anti- and pro-Angiogenic therapies. Genes Cancer.

[20] Hiratsuka S, Minowa O, Kuno J, Noda T, Shibuya M (1998). Flt-1 lacking the tyrosine kinase domain is sufficient for normal development and angiogenesis in mice. Proc Natl Acad Sci U S A.

[21] Kiba A, Sagara H, Hara T, Shibuya M (2003). VEGFR-2-specific ligand VEGF-E induces non-edematous hyper-vascularization in mice. Biochem Biophys Res Commun.

[22] Yuan J, Guo Q, Qureshi AR, Anderstam B, Eriksson M, Heimburger O, Barany P, Stenvinkel P, Lindholm B (2013). Circulating vascular endothelial growth factor (VEGF) and its soluble receptor 1 (sVEGFR-1) are associated with inflammation and mortality in incident dialysis patients. Nephrol Dial Transplant.

[23] Kauppinen A, Paterno JJ, Blasiak J, Salminen A, Kaarniranta K (2016). Inflammation and its role in age-related macular degeneration. Cell Mol Life Sci.

[24] Tolentino MJ, Miller JW, Gragoudas ES, Jakobiec FA, Flynn E, Chatzistefanou K, Ferrara N, Adamis AP (1996). Intravitreous injections of vascular endothelial growth factor produce retinal ischemia and microangiopathy in an adult primate. Ophthalmology.

